# Alleviation of Endoplasmic Reticulum Stress Enhances Human Corneal Epithelial Cell Viability under Hyperosmotic Conditions

**DOI:** 10.3390/ijms23094528

**Published:** 2022-04-20

**Authors:** Damien Guindolet, Ashley M. Woodward, Eric E. Gabison, Pablo Argüeso

**Affiliations:** 1 Schepens Eye Research Institute of Mass. Eye and Ear, Department of Ophthalmology, Harvard Medical School, 20 Staniford St., Boston, MA 02114, USA; damien.guindolet@gmail.com (D.G.); ashley.woodward@tufts.edu (A.M.W.); 2 Hôpital Fondation A. de Rothschild, 25 rue Manin, 75019 Paris, France; egabison@for.paris; 3 UFR Médecine, Université Paris Cité, 75018 Paris, France

**Keywords:** corneal epithelium, dry eye, endoplasmic reticulum stress, hyperosmotic stress, tauroursodeoxycholic acid, unfolded protein response

## Abstract

Tear hyperosmolarity plays an essential role in the initiation and progression of dry-eye disease. Under a hyperosmotic environment, corneal epithelial cells experience perturbations in endoplasmic reticulum function that can lead to proinflammatory signaling and apoptosis. In this study, we investigated the effect of tauroursodeoxycholic acid (TUDCA), a chemical chaperone known to protect against endoplasmic reticulum stress, on corneal epithelial cells exposed to hyperosmotic conditions. We found that the expression of the genes involved in the activation of the unfolded protein response and the pro-apoptotic transcription factor *DDIT3* were markedly upregulated in patients with Sjögren’s dry-eye disease and in a human model of corneal epithelial differentiation following treatment with hyperosmotic saline. Experiments in vitro demonstrated that TUDCA prevented hyperosmotically induced cell death by reducing nuclear DNA fragmentation and caspase-3 activation. TUDCA supplementation also led to the transcriptional repression of *CXCL8* and *IL5*, two inflammatory mediators associated with dry-eye pathogenesis. These studies highlight the role of hyperosmotic conditions in promoting endoplasmic reticulum stress in the cornea and identify TUDCA as a potential therapeutic agent for the treatment of dry-eye disease.

## 1. Introduction

Dry eye is a disorder of the ocular surface affecting millions of people worldwide and with limited therapeutic options [1]. In the U.S. alone, an estimated 3.2 million women aged 50 and older, and 1.68 million men age 50 and older, are affected by dry eye [2,3]. The core mechanism of the disease is evaporation-induced tear hyperosmolarity, which triggers a myriad of signaling events within surface epithelial cells and the release of proinflammatory mediators [4]. The combined action of hyperosmotic stress and inflammation is thought to cause epithelial cell damage and the loss of barrier function in the cornea and conjunctiva. Several lines of evidence support the notion that endoplasmic reticulum (ER) stress contributes to the pathogenesis of ocular drying disorders such as Sjögren’s syndrome [5], an autoimmune disease affecting the function of the lacrimal gland, mostly in women. However, the contribution of hyperosmolarity toward ER-stress-associated programmed cell death at the ocular surface epithelia has not been clearly established.

The ER is composed of a convoluted network of tubules and sacs that serve as the first compartment of the secretory pathway in eukaryotic cells [6]. It contains chaperones and folding enzymes that facilitate the assembly of newly synthesized proteins and prevent protein misfolding and aggregation. These proteins include the heat-shock protein family Member 5 (HSPA5), also known as BiP or GRP78, which is a chaperone that binds newly synthesized proteins as they are translocated into the ER and assists with their assembly. Hyperosmolar environments trigger ER stress, altering the trafficking of the secretory cargo in the ER and saturating its capacity to fold proteins [7]. The accumulation of unfolded proteins subsequently activates specific stress-signaling pathways, collectively known as the unfolded protein response (UPR), which transiently mitigate protein biosynthesis and increase ER protein-folding capacity. This phenomenon is associated with the activation of transcription factors, such as sXBP1, that direct the expression of chaperones and folding enzymes aimed at promoting cell adaptation and restoring homeostasis. In cases where severe ER stress conditions persist, the UPR initiates apoptosis to remove the stressed cells in a process largely mediated by DNA-damage-inducible transcript 3 protein (DDIT3), also known as CHOP [8].

Recently, much progress has been made in identifying the small molecules, known as chemical chaperones, that improve ER folding capacity and facilitate the trafficking of proteins through the secretory pathway [9]. One of these molecules, tauroursodeoxycholic acid or TUDCA, is a bile acid derivative used for centuries in Chinese medicine with potential therapeutic benefits for diabetes, obesity, and neurodegeneration [10,11]. Structure–function analyses have demonstrated that TUDCA inhibits the aggregation of thermally denatured proteins at millimolar concentrations through direct interactions with other molecular chaperones or secondary to the direct stabilization of the proteins [12]. TUDCA has been demonstrated to promote cytoprotective activities by suppressing apoptosis and decreasing inflammation in many models of human disease [10]. In the present study, we identify the function of TUDCA in preventing cell death in human corneal epithelial cell cultures exposed to hyperosmolar conditions. Mechanistically, we demonstrate that TUDCA reduces nuclear DNA fragmentation and caspase-3 activation and leads to the transcriptional repression of the inflammatory mediators associated with dry eye pathogenesis. These data highlight the therapeutic value of chemical chaperones targeting ER stress as an alternative to the anti-inflammatory modalities, such as lifitegrast and cyclosporine, that are currently used to restore epithelial cell homeostasis in patients with dry eye disease.

## 2. Results

### 2.1. DDIT3 Is Upregulated in Sjögren’s Syndrome

**Figure 1:**
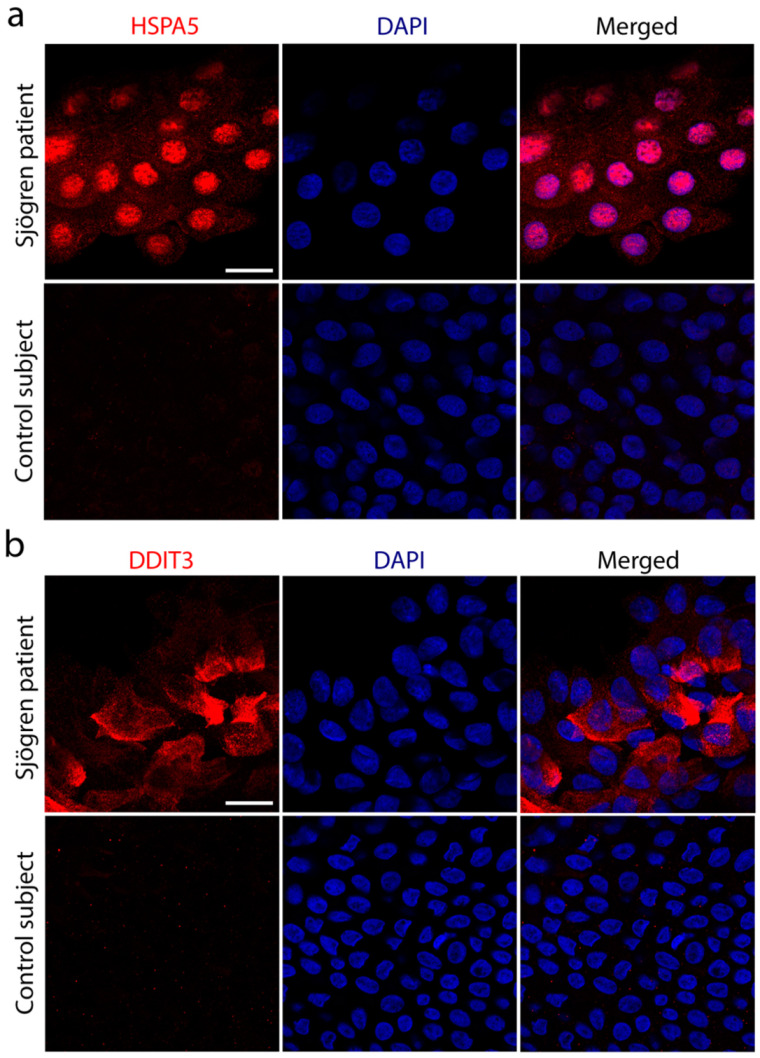
Expression of indicators of cells undergoing ER stress in conjunctival epithelium of patients with Sjögren’s syndrome and control subjects. Conjunctival impression cytology samples were collected from the superior and temporal bulbar conjunctiva. Cells in the filters were immunostained with antibodies against HSPA5 (**a**) or DDIT3 (**b**), and counterstained with DAPI. Scale bars: 20 µm.

Previous observations by Coursey et al. found elevated levels of HSPA5 and sXBP1 in the conjunctival epithelium of Sjögren’s syndrome patients, which were linked to the presence of ER stress and the induction of the UPR in mucin-secreting goblet cells [5]. Our experiments confirmed the presence of HSPA5 in the conjunctival epithelium of Sjögren’s syndrome patients but, additionally, they showed the nuclear translocation of HSPA5 (Figure 1a and Supplementary Figure S1). We were not surprised by this observation because HSPA5 was previously localized to the nucleus following the induction of ER stress, when it might play a role against apoptotic events induced by DNA damage [13]. To gain further knowledge about the balance between survival and apoptosis signaling factors in Sjögren’s syndrome conjunctiva, we evaluated the expression of the pro-apoptotic factor DDIT3. In these experiments, we observed an accumulation of DDIT3 in conjunctival epithelial cells from pathological specimens compared to the control (Figure 1b and Supplementary Figure S1), indicating that severe ER stress and epithelial damage at the ocular surface of Sjögren’s syndrome patients is associated with the activation of the DDIT3 signaling pathway.

### 2.2. Hyperosmolarity Induces the UPR in Stratified Human Corneal Epithelial Cells

**Figure 2:**
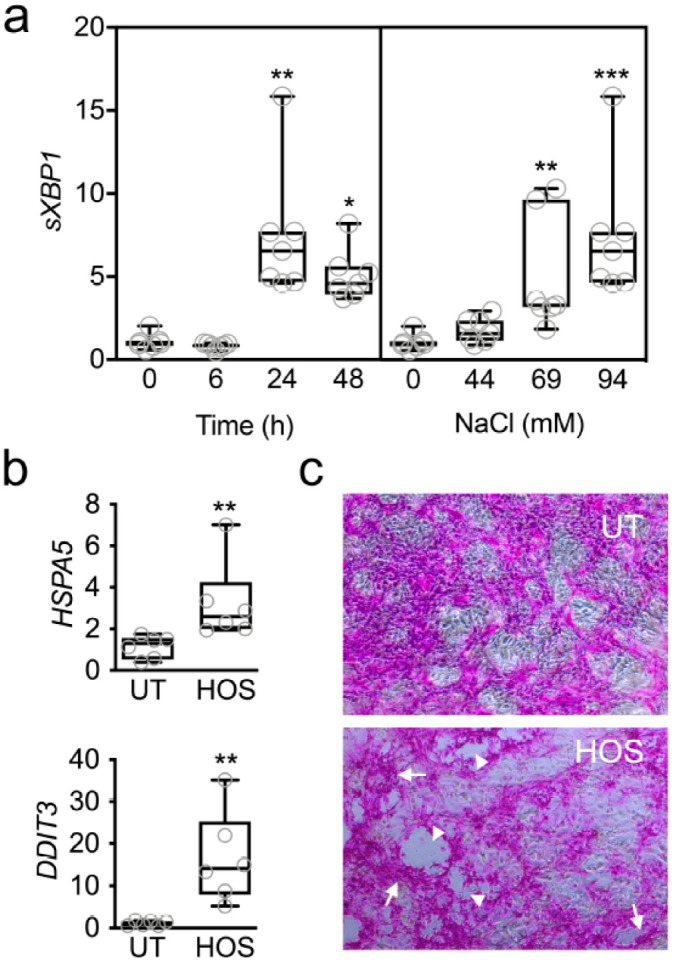
Hyperosmolarity induces the UPR in stratified human corneal epithelial cells. (**a**) Cell cultures exposed to sodium chloride were evaluated for time- and concentration-dependent changes in *sXBP1* expression. qPCR experiments demonstrated significant upregulation of *sXBP1* following addition of sodium chloride (94 mM) for 24–48 h and using 69–94 mM sodium chloride (for 24 h) (*n* = 7 independent experiments). (**b**) *HSPA5* and *DDIT3* mRNA were significantly upregulated by qPCR following exposure to HOS (94 mM NaCl, 24 h) compared to untreated (UT) cells (*n* = 6 independent experiments). (**c**) HOS led to the formation of areas with substantial cell loss (arrowheads) and the penetration of rose bengal into damaged cells with altered morphology (arrows). The box-and-whisker plots show the 25th and 75th percentiles (boxes), the median, and the minimum and maximum data values (whiskers). Significance was determined using the Kruskal–Wallis test followed by Dunn’s multiple comparison (**a**) or Mann–Whitney test (**b**). *, *p* < 0.05; **, *p* < 0.01; ***, *p* < 0.001.

To evaluate whether hyperosmolarity affects the UPR at the ocular surface, we used a human model of corneal epithelial cell differentiation that mimics the characteristics of the native epithelium in terms of stratification and barrier function [14,15]. The exposure of these cell cultures to hyperosmotic saline led to a time- and concentration-dependent increase in *sXBP1* transcription (Figure 2a). We found that the expression levels of the *sXBP1* peaked 24 h after the addition of sodium chloride, with the highest concentration of 94 mM leading to the greatest upregulation of mRNA levels. Consequently, in subsequent experiments, we treated the cell cultures with 94 mM sodium chloride for 24 h to induce hyperosmolar stress (HOS). Under these conditions, we found that the expressions of *HSPA5* and *DDIT3* were significantly upregulated (Figure 2b). The activation of the UPR in these cells was concomitant with the increased staining with rose bengal, an organic anionic dye used to assess damage to the ocular surface epithelium in dry-eye disease. In these experiments, HOS led to the formation of areas with substantial cell loss and the penetration of the dye into damaged cells with altered morphology (Figure 2c).

### 2.3. Alleviation of ER Stress Reduces Hyperosmotically Induced Cell Death

**Figure 3:**
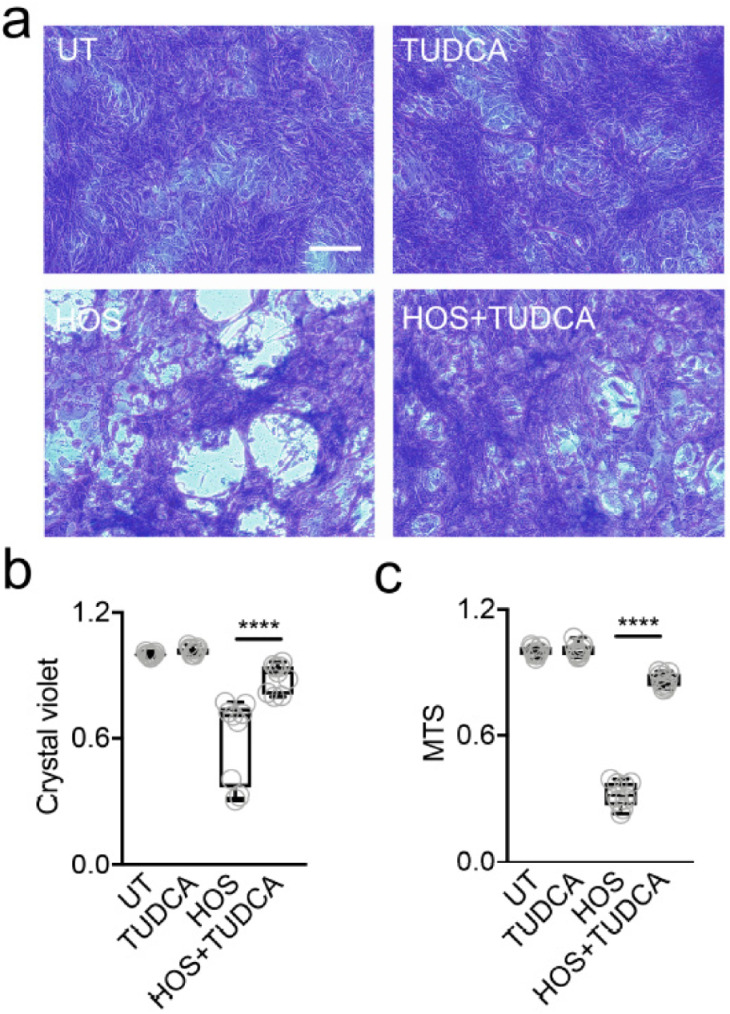
TUDCA reduces hyperosmotically induced cell death. (**a**) Crystal violet staining of stratified human corneal epithelial cells: untreated (UT), treated with 1 mM TUDCA alone (TUDCA), or exposed to HOS (94 mM NaCl, 24 h) with or without addition of TUDCA. Scale bar: 10 µm. (**b**) Quantitative assessment of the cellular area covered by crystal violet in (**a**) normalized-to-UT-control (*n* = 3 independent experiments in triplicate). (**c**) Quantitative assessment of cell viability using the MTS assay (*n* = 3 independent experiments in triplicate). The box-and-whisker plots show the 25th and 75th percentiles (boxes), the median, and the minimum and maximum data values (whiskers). Significance was determined using the Kruskal–Wallis test followed by Dunn’s multiple comparison. ****, *p* < 0.0001.

Accumulating evidence indicates that chemical chaperones can prevent ER-stress-associated programmed cell death after the prolonged activation of the UPR [16]. Therefore, we assessed whether supplementation with the chemical chaperone TUDCA could prevent the adverse effects of hyperosmolarity in stratified human corneal epithelial cells. In these experiments, we made use of crystal violet, a dye commonly used to assess viability in adherent cell culture models [17]. As expected, the exposure of the stratified cell cultures to HOS led to a clear loss of cell biomass (Figure 3a,b). On the other hand, the supplementation of the hyperosmotic culture media with 1 mM TUDCA resulted in a significant reduction in cell loss. These data were confirmed by measuring the cellular dehydrogenase activity with the MTS assay, an indicator of the number of viable cells in culture systems (Figure 3c).

**Figure 4:**
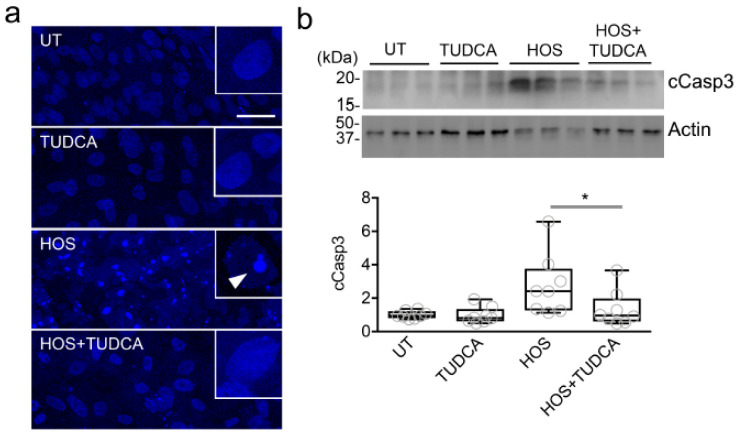
TUDCA reduces hyperosmotically induced apoptotic events. (**a**) DAPI staining showing increased chromatin condensation and fragmentation (arrowhead in the inset) in stratified human corneal epithelial cells exposed to HOS (94 mM NaCl, 24 h) compared to control cells (UT, TUDCA alone) or cells exposed to HOS in the presence of TUDCA. UT, untreated cells. Scale bar: 50 µm. (**b**) Upper panel: Western blotting analysis showing cleaved caspase-3 (cCasp3) and ß-actin. Molecular weights of standard proteins are shown to the left. Lower panel: Quantification of the relative levels of cCasp3 protein in cell culture (*n* = 3 independent experiments in duplicate or triplicate). The box-and-whisker plots show the 25th and 75th percentiles (boxes), the median, and the minimum and maximum data values (whiskers). Significance was determined using the Kruskal–Wallis test followed by Dunn’s multiple comparison. *, *p* < 0.05.

We further assessed whether the supplementation with TUDCA was associated with a reduction in apoptotic events. First, we used DAPI staining to examine the chromatin condensation and fragmentation in the nuclei of the cells undergoing apoptosis, as previously reported [18]. We found that the stratified corneal epithelial cells not subjected to HOS were characterized by a homogeneous distribution of DAPI staining in their nuclei (Figure 4a). Conversely, the exposure to hyperosmotic saline led to increased DAPI intensity, corresponding to areas of chromatin condensation and fragmentation. Importantly, these alterations in nuclear morphology were greatly reduced in the presence of TUDCA. Second, we evaluated the activation of caspase-3, which requires cleavage into smaller subunits under apoptotic conditions and is activated under hyperosmotic conditions in human corneal epithelial cells, as a surrogate marker of ER-stress-associated programmed cell death [19,20,21]. We found that HOS led to the increased cleavage of caspase-3, but that this effect could be significantly blocked by TUDCA supplementation (Figure 4b). These results strengthen the concept that TUDCA can enhance the survival of corneal epithelial cells via reductions in apoptosis.

### 2.4. TUDCA Downregulates CXCL8 and IL5 under Hyperosmotic Conditions

**Figure 5:**
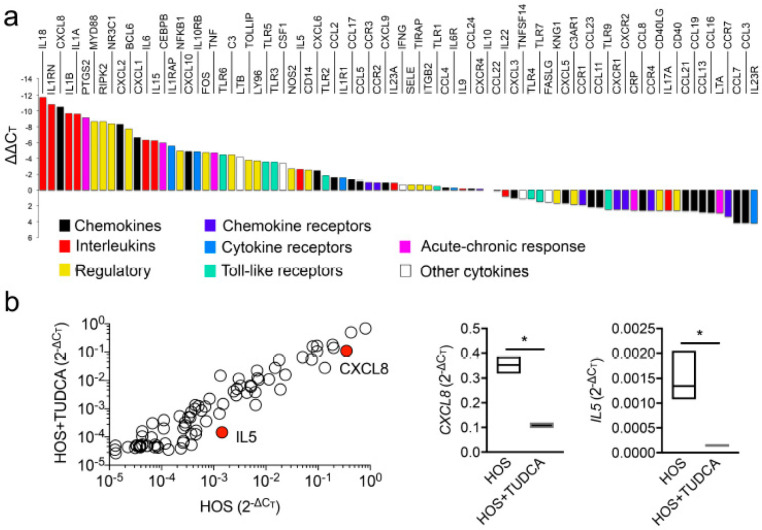
TUDCA downregulates *CXCL8* and *IL5* under hyperosmotic conditions. Stratified human corneal epithelial cells were exposed to HOS (94 mM NaCl, 24 h) with or without addition of 1 mM TUDCA. (**a**) Relative transcript abundance of genes encoding proteins involved in the inflammatory response in cells exposed to HOS. The expression of genes was normalized using the comparative ∆∆C_T_ method. (**b**) Scatterplot comparing the expression of genes in cells exposed to HOS with or without TUDCA (*n* = 3 independent experiments). The red dots indicate statistically significant downregulation of the immune regulatory gene. The corresponding quantitative graphs are shown to the right. The data are presented as floating bars (min. to max.) with line at the median. Significance was determined using the unpaired *t* test with a Holm–Sidak’s multiple comparison. *, *p* < 0.05.

Tear hyperosmolarity damages the ocular surface epithelia both directly and indirectly, through a cascade of inflammatory events [22]. We used a PCR array to investigate the ability of TUDCA to modulate the expression of a focused panel of genes involved in the proinflammatory response following exposure to HOS. Consistent with previous reports, a direct comparison of the relative expression levels in epithelial cells exposed to HOS revealed that the most highly expressed genes included interleukins (IL1, IL5, IL6, and TNFα) and chemokines such as CXCL8 and CXCL10 (Figure 5a), which are commonly observed in patients with dry-eye disease [23]. Other genes that were prevalently expressed, and associated with the pathogenesis of dry eye, included the chemokine receptor CCR2 and the T-cell chemoattractant, CCL5. Two genes were found to be significantly downregulated in cell cultures incubated with TUDCA, including CXCL8, a neutrophil chemoattractant involved in the innate immune response, and IL5, a pleiotropic cytokine commonly associated with eosinophil survival (Figure 5b).

## 3. Discussion

Tear hyperosmolarity, commonly accepted as a core mechanism in the development of dry eye, can lead to epithelial cell death and trigger a myriad of inflammatory responses at the ocular surface. Many cells cope with hyperosmolarity through physiological processes such as the regulation of cell volume and the activation of osmoprotective signaling pathways aimed at restoring homeostasis [24]. During the past decade, ER stress and the activation of the UPR have emerged as important regulatory components in the initiation, progression or resolution of the pathological responses observed in dry-eye disease [5,25]. However, the contribution of hyperosmolarity to these processes remains far less understood. In this study, we used a human model of corneal epithelial differentiation to demonstrate that hyperosmotic conditions trigger the expression of the genes involved in ER stress and the activation of the UPR. We show that the alleviation of ER stress with the chemical chaperone TUDCA prevents cell death by reducing nuclear DNA fragmentation and caspase-3 activation, and represses the transcription of two inflammatory mediators, *CXCL8* and *IL5*, that are associated with dry-eye pathogenesis.

Hyperosmolarity has been linked to ER stress and the induction of cell death in multiple cell types, such as cardiac myocytes, neurons, and oocytes [26,27,28]. Studies in rabbits have demonstrated that applying high osmotic pressure to subconfluent cultures of corneal epithelial cells leads to the production of reactive oxygen species, the activation of the ER signaling pathway and, ultimately, cell apoptosis [29]. Our study in patients with Sjögren’s syndrome, and a stratified human model of corneal epithelial differentiation, supports these findings and points to the involvement of the pro-apoptotic factor DDIT3 in mediating epithelial cell death. DDIT3 is known to induce cell death by directly activating GADD34, a DNA-damage-inducible protein expressed in cells exposed to ER stress [30]. GADD34 activation results in protein synthesis recovery, which further exacerbates ER stress and the initiation of apoptotic events. Unexpectedly, the induction of GADD34 has been found to allow adaption to hyperosmotic stress in cultures of SV40-transformed human corneal epithelial cells [31]. The osmoadaptative properties of GADD34 in this model have been attributed to its ability to reverse hyperosmotic-stress-induced Golgi fragmentation and the maintenance of the microtubule network. This adaptation is characterized by the fast processing and trafficking of the SNAT2 transporter through the Golgi complex, which facilitates the uptake of compatible osmolytes [31]. It is important to note, however, that these protective properties of GADD34 were observed under mild, but not severe, hyperosmotic conditions, the latter significantly contributing to corneal inflammation and the more serious complications found in patients with dry-eye disease [32].

The failure of cells to restore protein-folding homeostasis after the prolonged activation of the UPR leads to a signaling switch that favors apoptosis. At the ocular surface, apoptosis has been linked to the pathogenesis of dry-eye disease and has been identified as a therapeutic target for this condition [33]. In our experiments, we found that TUDCA prevented hyperosmotically induced cell death by reducing nuclear DNA fragmentation and caspase-3 activation, indicating that chemical chaperones could be therapeutically used to alleviate ER stress, reduce apoptosis, and promote epithelial cell homeostasis in dry-eye disease. This premise is supported by the ability of TUDCA to reduce apoptosis in multiple tissues and under different pathological states, including retinal diseases [10,34]. Although our in vitro model system allowed us to evaluate the protective function of TUDCA under a particular stress condition (i.e., hyperosmolarity), a potential limitation related to the present work is the lack of an in vivo model of dry-eye disease that could shed additional light on the therapeutic implications of TUDCA supplementation in a more complex environment. Mechanistically, TUDCA not only mitigates caspase-3 activation but also influences additional signaling pathways in both epithelial and non-epithelial cells [10]. For instance, TUDCA has been shown to inhibit apoptosis by preserving mitochondrial membrane stability in neurons and to decrease the release of inflammatory mediators by immune cells [35,36]. Therefore, in vivo models should be developed in further studies to determine the multiple actions of TUDCA at the ocular surface.

One interesting finding in this report is the ability of TUDCA to reduce the expression of *CXCL8* produced by corneal epithelial cells under hyperosmotic conditions. The *CXCL8* gene encodes the IL8 protein, one of the most intensively studied chemokines involved in the activation and trafficking of immune cells, including neutrophils. IL8 has been consistently detected in the tear fluid and conjunctival epithelia of patients with dry-eye disease [37,38]. The literature indicates that IL8 levels correlate with pain and with clinical parameters related to tear stability and ocular surface integrity [39]. Since the increased expression of *CXCL8* by resident tissues is an important mechanism for directing inflammation, it has been proposed that targeting IL8 could constitute a promising therapeutic approach for dry-eye patients [38]. In this regard, TUDCA might constitute an effective drug, based on its ability to decrease *CXCL8* expression. Interestingly, our results also indicate that TUDCA reduces the expression of *IL5*, a major maturation and differentiation factor for eosinophils, although the significance of this finding for the pathophysiology of dry eye remains unclear. We believe that future experiments with additional stressors of the ocular surface, other than hyperosmolarity, should provide further insight into the involvement of TUDCA in the resolution of inflammatory processes. Altogether, our work indicates that targeting ER stress by using a chemical chaperone attenuates hyperosmotically induced cell death in human corneal epithelial cells and could have important therapeutic implications for restoring homeostasis at the ocular surface.

## 4. Materials and Methods

### 4.1. Human Samples

This study was conducted in accordance with the tenets of the Declaration of Helsinki. The research was approved by the Rothschild Foundation Hospital Review Board, IRB 00012801, under study number NCT03358979. Written informed consent was obtained from all the participants. Samples were obtained from six female patients (ages 42 to 65 years) diagnosed with Sjögren’s syndrome and an ocular surface staining grade of at least III based on the standard Oxford scale [40]. Healthy controls were obtained from six female subjects (aged 24 to 33 years) who had no corneal or conjunctival fluorescein staining. Conjunctival impression cytology samples were collected using a 20-micrometer polyethersulfone filter (Pall Laboratories Supor Membrane filters, Ann Arbor, MI, USA) that was applied with slight pressure to the superior and temporal bulbar conjunctiva after topical anesthesia. After five seconds, filters were removed and placed in tubes filled with a 4% paraformaldehyde solution (Merck, Darmstadt, Germany) for 20 min at room temperature. Next, the filters were rinsed with distilled water and stored at −80 °C until they were processed for immunofluorescence.

### 4.2. Cell Culture

Telomerase-immortalized human corneal epithelial cells were grown as previously reported [41]. Briefly, cells were plated in 12-well plates (Costar Corning, Corning, NY, USA) at a seeding density of 2.5 × 10^4^ cells/cm^2^ and maintained in keratinocyte serum-free medium (Thermo Fisher Scientific, Waltham, MA, USA) at 37 °C in 5% CO_2_ until confluence. Subsequently, cells were grown in Dulbecco’s Modified Eagle’s medium/F12 supplemented with 10% calf serum (Hyclone-GE Healthcare Life Sciences, Marlborough, MA, USA), 1% penicillin-streptomycin (Thermo Fisher Scientific), and 10 ng/mL epidermal growth factor (PHG0311, Thermo Fisher Scientific) for 7 days to promote stratification and differentiation [14]. Next, cells were cultured for an additional 50 h in an equal volume of serum-free media. To increase osmolarity, a sterile solution of sodium chloride was added 6, 24 or 48 h before the end of the experiment, to generate an additional concentration of 0, 44, 69 or 94 mM sodium chloride, as reported elsewhere [42]. When specified, 1 mM of TUDCA (Millipore Sigma, Burlington, MA, USA) was added along with the sodium chloride.

### 4.3. qPCR

Total RNA was isolated from cell cultures using TRIzol (Thermo Fisher Scientific) following the manufacturer’s instructions. Residual genomic DNA was eliminated by DNase I digestion (Thermo Fisher Scientific) of the RNA preparation and then 1 µg of total RNA, transcribed using the iScript^TM^ cDNA synthesis kit (Bio-Rad, Hercules, CA, USA). PCR was performed using the SsoAdvanced™ Universal SYBR^®^ Green Supermix (Bio-Rad). Primer sequences for *sXBP1* (forward 5′-CTGAGTCCGAATCAGGTGCAG-3′; reverse 5′-ATCCATGGGGAGATGTTCTGG-3′) and *DDIT3* (forward 5′-AGAACCAGGAAACGGAAACAGA-3′; reverse 5′-TCTCCTTCATGCGCTGCTTT-3′) mRNA have been published previously [43,44]. Primer sequences for *HSPA5* (unique assay ID qHsaCID0012663) and *ACTB* (unique assay ID qHsaCEP0036280) mRNA were obtained from Bio-Rad. Gene expression was measured in a Mastercycler ep realplex thermal cycler (Eppendorf, Hauppauge, NY, USA) with the following parameters: 2 min at 95 °C, followed by 40 cycles of 5 s at 95 °C and 30 s at 60 °C. Fold changes were calculated using the comparative ∆∆C_T_ method by normalizing to *ACTB*.

### 4.4. RT^2^ Profiler PCR Array

The Human Inflammatory Response & Autoimmunity RT^2^ Profiler PCR array (Qiagen, PAHS-077Z) was used according to the manufacturer’s instructions. A total of 84 genes involved in the inflammatory response were included in the array. Three independent experiments were performed using 1 µg of cDNA of starting material, comparing cells exposed for 24 h to 94 mM sodium chloride with or without 1 mM TUDCA. Data were analyzed using web-based PCR array data analysis software (SABiosciences, Frederick, MD, USA).

### 4.5. Crystal Violet Assay

Cells were fixed with 100% methanol for 5 min at room temperature and then stained with 0.1% crystal violet (Sigma-Aldrich, St. Louis, MO, USA) in 50% methanol. After washing with PBS, cells were photographed with a 10X objective using an Eclipse TS100 inverted microscope (Nikon, Tokyo, Japan). The stained area was quantified using ImageJ software (NIH, Bethesda, MD, USA) and data were normalized to untreated cells.

### 4.6. Rose Bengal Staining

Transcellular barrier function was assayed with the rose bengal anionic dye, as previously described [41]. Cell cultures were rinsed with PBS and incubated for five minutes with a 0.1% solution of rose bengal (Thermo Fisher Scientific) in PBS. Subsequently, the dye was aspirated and the culture washed with PBS. The extent of dye penetrance was assessed using an inverted microscope (Nikon Eclipse TS100) and the images were analyzed using ImageJ software.

### 4.7. MTS Assay

Cell viability was measured using the CellTiter 96^®^ AQueous One Solution Cell Proliferation Assay (Promega, Madison, WI, USA). Briefly, 200 µL of MTS reagent was added to each well in the cell culture and incubated for 2 h at 37 °C. Next, a total of 100 µL of the culture media was transferred into a 96-well plate in triplicate. Absorbance was measured using a microplate reader (SpectraMax Plus; Molecular Devices; San Jose, CA, USA) with a wavelength of 495 nm. Relative fold change was generated by normalizing absorbance values to untreated controls.

### 4.8. Western Blotting

Total cellular protein was extracted using RIPA buffer (150 mM NaCl, 50 mM Tris, pH 8.0, 1% NP 40, 0.5% deoxycholate, 0.1% SDS) supplemented with cOmplete™ Protease Inhibitor Cocktail (Roche Diagnostics, Indianapolis, IN). After homogenization with a pellet pestle, the protein cell extracts were centrifuged at 12,000× *g* for 45 min, and the protein concentration of the supernatant was determined using the Pierce BCA Protein Assay Kit (Thermo Fisher Scientific). Proteins in cell lysates (25 μg) were resolved in 15% SDS–PAGE and electroblotted onto nitrocellulose membranes (Bio-Rad). Nonspecific binding to the membranes was blocked by incubation with 5% bovine serum albumin in Tris-buffered saline containing 0.1% Tween-20 (TTBS) for 1 h at room temperature. Next, membranes were incubated with anti-caspase-3 (1:1000; #9662; Cell Signaling Technology) or anti-ß-actin (1:1000; #3700; Cell Signaling Technology) antibodies in 5% bovine serum albumin in TTBS overnight at 4 °C, followed by the corresponding peroxidase-conjugated anti-rabbit IgG secondary antibody (1:3000; sc-2357; Santa Cruz Biotechnology, Paso Robles, CA, USA) in 5% bovine serum albumin in TTBS for 1 h at room temperature. Peroxidase activity was detected on HyBlot CL autoradiography film (Denville Scientific, Plainfield, NJ, USA). Band intensities were quantified by densitometry using ImageJ software.

### 4.9. Immunofluorescence

Filters containing the human conjunctival specimens were hydrated for 5 min with distilled water, permeabilized and blocked in a PBS solution containing 5% bovine serum albumin, 0.5% Tween-20 and 1% Triton X-100 for 1 h at room temperature. The cells were then incubated with anti-HSPA5 (1:100; #610978; BD Biosciences, Franklin Lakes, NJ, USA) or anti-DDIT3 (1:100; sc-7351; Santa Cruz Biotechnology) antibodies overnight at 4 °C. Samples were washed and incubated with the corresponding Alexa Fluor 555-conjugated secondary antibody (1:500; Thermo Fisher Scientific) and DAPI for 1 h at room temperature. Samples were mounted with a drop of Permafluor mounting medium (Thermo Fisher Scientific) and visualized using a Zeiss LSM 800 confocal microscope (Carl Zeiss MicroImaging GmbH, Jena, Germany). For cell culture experiments, cells were grown on glass chamber slides, washed with Dulbecco’s PBS, and fixed with 4% paraformaldehyde for 30 min. The slides were then coverslipped with antifade mounting medium containing DAPI (Vectashield; Vector Laboratories, Burlingame, CA, USA) and imaged using a Leica TCS SP5 confocal microscope (Leica Microsystems, Wetzlar, Germany).

### 4.10. Statistics

Statistical analyses were performed using Prism software version 9 (GraphPad Software, San Diego, CA, USA) for Macintosh. Significance was determined using the Kruskal–Wallis test followed by Dunn’s multiple comparison, the Mann–Whitney test, or the unpaired *t* test with a Holm–Sidak’s multiple comparison.

## Supplementary Materials

The following supporting information can be downloaded at: https://www.mdpi.com/article/10.3390/ijms23094528/s1.
