# Supplementary material for: Alleviation of Endoplasmic Reticulum Stress Enhances Human Corneal Epithelial Cell Viability under Hyperosmotic Conditions
Source: Int J Mol Sci. 2022 Apr 20;23(9):4528. doi: 10.3390/ijms23094528 (PMC9104051; doi:10.3390/ijms23094528)

Supplementary Figure S1. Expression of indicators of cells undergoing ER stress in conjunctival epithelium of additional patients with Sjögren's syndrome and control subjects. Cells in the filters were immunostained with antibodies against HSPA5 or DDIT3, and counterstained with DAPI. Scale bars: 20 μm.

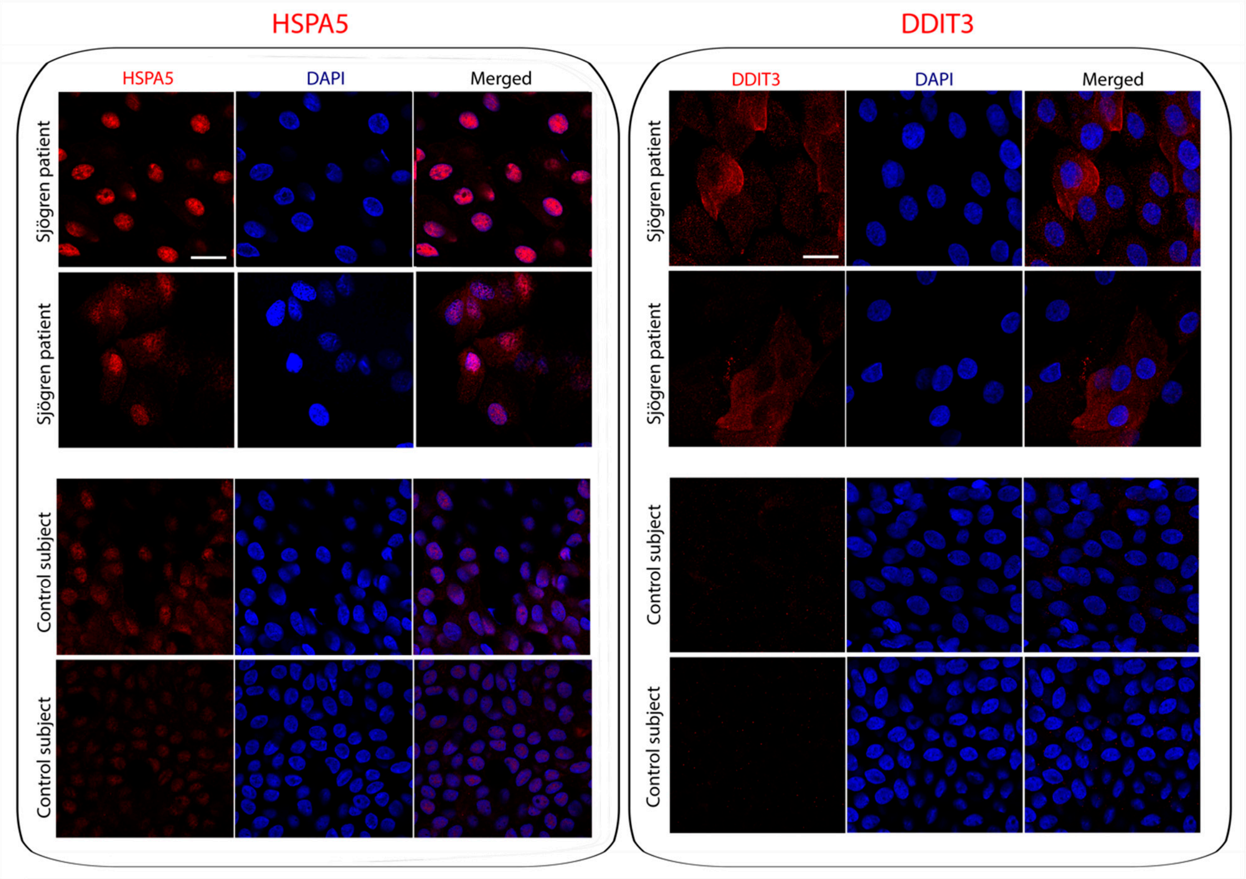

Supplement: Supplementary file 1 [file ijms-23-04528-s001.zip › Supplementary Figure S1.pdf]
