# Supplementary material for: Alleviation of Endoplasmic Reticulum Stress Enhances Human Corneal Epithelial Cell Viability under Hyperosmotic Conditions
Source: Int J Mol Sci. 2022 Apr 20;23(9):4528. doi: 10.3390/ijms23094528 (PMC9104051; doi:10.3390/ijms23094528)
Supplement: Supplementary file 1 [file ijms-23-04528-s001.zip › Supplementary Table S1.pdf]

Supplementary Table S1. Relative expression of genes responsible for inflammation and autoimmunity in stratified human corneal epithelial cells. Cell cultures were exposed to HOS (94 mM NaCl, 24 h). Values in bold indicate statistically significant changes in gene expression following addition of 1 mM TUDCA. High positive ΔC_T values reflect low amplification efficiency.

Gene Symbol	RefSeq*	Magnitude of Expression (ΔC_T)	HOS+TUDCA (fold change)
BCL6	NM_001706	4.30	1.30
C3	NM_000064	7.61	1.30
C3AR1	NM_004054	13.87	4.57
CCL2	NM_002982	10.45	0.50
CCL3	NM_002983	16.22	3.73
CCL4	NM_002984	11.77	0.90
CCL5	NM_002985	10.94	2.45
CCL7	NM_006273	16.21	2.02
CCL8	NM_005623	14.64	1.24
CCL11	NM_002986	14.23	0.94
CCL13	NM_005408	14.87	1.45
CCL16	NM_004590	14.90	1.52
CCL17	NM_002987	10.75	1.51
CCL19	NM_006274	14.70	1.21
CCL21	NM_002989	14.70	1.18
CCL22	NM_002990	12.14	2.45
CCL23	NM_005064	14.18	0.90
CCL24	NM_002991	11.88	0.78
CCR1	NM_001295	13.93	1.51
CCR2	NM_001123396	11.11	0.27
CCR3	NM_001837	11.09	0.37
CCR4	NM_005508	14.64	1.10
CCR7	NM_001838	15.43	1.93
CD14	NM_000591	9.54	11.06
CD40	NM_001250	14.66	1.24
CD40LG	NM_000074	14.64	1.11
CEBPB	NM_005194	6.06	1.87
CRP	NM_000567	14.63	1.23

CSF1	NM_000757	8.69	1.71
CXCL1	NM_001511	5.40	0.68
CXCL2	NM_002089	3.73	0.74
CXCL3	NM_002090	13.06	0.50
CXCL5	NM_002994	13.72	0.58
CXCL6	NM_002993	9.60	0.53
CXCL8	**NM_000584**	**1.51**	**0.31**
CXCL9	NM_002416	11.14	3.04
CXCL10	NM_001565	7.18	1.75
CXCR1	NM_000634	14.52	3.75
CXCR2	NM_001557	14.52	2.47
CXCR4	NM_003467	11.91	0.24
FASLG	NM_000639	13.57	0.59
FOS	NM_005252	7.30	0.22
IFNG	NM_000619	11.38	0.14
IL1A	NM_000575	2.40	0.95
IL1B	NM_000576	2.33	0.71
IL1R1	NM_000877	10.51	1.75
IL1RAP	NM_002182	6.45	0.95
IL1RN	NM_000577	1.22	1.15
IL5	**NM_000879**	**9.44**	**0.10**
IL6	NM_000600	5.72	0.25
IL6R	NM_000565	11.78	1.17
IL9	NM_000590	11.88	0.27
IL10	NM_000572	12.05	0.17
IL10RB	NM_000628	7.20	3.29
IL15	NM_000585	5.80	2.92
IL17A	NM_002190	14.64	1.31
IL18	NM_001562	0.30	0.85
IL22	NM_020525	12.86	0.26
IL23A	NM_016584	11.16	2.25
IL23R	NM_144701	16.26	2.77
ITGB2	NM_000211	11.47	1.39
KNG1	NM_000893	13.71	0.71

LTA	NM_000595	14.97	1.31
LTB	NM_002341	7.86	0.86
LY96	NM_015364	8.37	3.82
MYD88	NM_002468	3.38	1.74
NFKB1	NM_003998	7.10	1.46
NOS2	NM_000625	9.37	0.98
NR3C1	NM_000176	3.66	2.00
PTGS2	NM_000963	2.89	0.21
RIPK2	NM_003821	3.39	1.09
SELE	NM_000450	11.39	0.35
TIRAP	NM_001039661	11.40	2.06
TLR1	NM_003263	11.58	1.53
TLR2	NM_003264	10.23	2.70
TLR3	NM_003265	8.53	1.10
TLR4	NM_138554	13.15	1.83
TLR5	NM_003268	8.48	1.81
TLR6	NM_006068	7.59	1.16
TLR7	NM_016562	13.52	0.49
TLR9	NM_017442	14.51	1.25
TNF	NM_000594	7.32	0.62
TNFSF14	NM_003807	13.15	3.59
TOLLIP	NM_019009	8.25	2.00

*Reference sequence database at NCBI (https://www.ncbi.nlm.nih.gov/refseq/).
